# The protective effect of WKYMVm peptide on inflammatory osteolysis through regulating NF‐κB and CD9/gp130/STAT3 signalling pathway

**DOI:** 10.1111/jcmm.14885

**Published:** 2019-12-14

**Authors:** Junxian Hu, Xianghe Li, Yueqi Chen, Xinyun Han, Li Li, Zhengwei Yang, Lianli Duan, Hongwei Lu, Qingyi He

**Affiliations:** ^1^ Department of Orthopedics Southwest Hospital Army Medical University (Third Military Medical University) Chongqing China; ^2^ Guizhou Medical University Guiyang China; ^3^ Institute for Clean Energy & Advanced Materials Faculty of Materials and Energy Southwest University Chongqing China; ^4^ Department of Orthopedics The First Affiliated Hospital of Zhengzhou University Zhengzhou China

**Keywords:** inflammatory osteolysis, lipopolysaccharide, osteoclastogenesis, WKYMVm peptide

## Abstract

The balance between bone formation and bone resorption is closely related to bone homeostasis. Osteoclasts, originating from the monocyte/macrophage lineage, are the only cell type possessing bone resorption ability. Osteoclast overactivity is thought to be the major reason underlying osteoclast‐related osteolytic problems, such as Paget's disease, aseptic loosening of prostheses and inflammatory osteolysis; therefore, disruption of osteoclastogenesis is considered a crucial treatment option for these issues. WKYMVm, a synthetic peptide, which is a potent FPR2 agonist, exerts an immunoregulatory effect. This peptide inhibits the production of inflammatory cytokines, such as (IL)‐1β and TNF‐α, thus regulating inflammation. However, there are only few reports on the role of WKYMVm and FPR2 in osteoclast cytology. In the current study, we found that WKYMVm negatively regulates RANKL‐ and lipopolysaccharide (LPS)‐induced osteoclast differentiation and maturation in vitro and alleviates LPS‐induced osteolysis in animal models. WKYMVm down‐regulated the expression of osteoclast marker genes and resorption activity. Furthermore, WKYMVm inhibited osteoclastogenesis directly through reducing the phosphorylation of STAT3 and NF‐kB and indirectly through the CD9/gp130/STAT3 pathway. In conclusion, our findings demonstrated the potential medicinal value of WKYMVm for the treatment of inflammatory osteolysis.

## 1 INTRODUCTION

The balance between bone resorption and bone formation is crucial for bone matrix homeostasis and human health.^1^ Osteoclast formation contributes to many osteolytic problems, including osteoporosis, Paget's disease, aseptic loosening of prostheses and inflammatory osteolysis, which may increase the fracture risk. Therefore, the treatment of these osteolytic diseases may depend on the inhibition of o osteoclastogenesis.^2^, ^3^

Osteoclasts exert their bone resorption activity mainly after the formation of mature osteoclasts.^4^ Osteoclastogenesis is a complex process involving various cytokines and the cell–cell fusion of osteoclast precursors. There are two ubiquitous and indispensable cytokines for early preosteoclast differentiation during the formation of mature osteoclasts, namely the receptor activator of nuclear factor‐κ B ligand (RANKL) and macrophage‐colony stimulating factor (M‐CSF). RANKL and M‐CSF are crucial not only for proliferation and differentiation but also for the fusion of the osteoclast precursors into functional multinucleated giant cells.^5^, ^6^, ^7^ When RANKL binds to its receptor RANK, the tumour necrosis factor (TNF) receptor–associated factor 6 (TRAF6) is recruited by osteoclast precursors, known as tartrate‐resistant acid phosphatase (TRAP)‐mononuclear macrophages. Subsequently, the nuclear factor‐kappa B (NF‐κ B) and mitogen‐activated protein kinases (MAPKs) signalling pathways are activated and further promote the nuclear translocation of NFATc1 and c‐Fos.^8^, ^9^, ^10^, ^11^ Thereafter, downstream marker osteoclast genes, such as matrix metalloproteinase 9 (MMP9), cathepsin K (CTSK) and tartrate‐resistant acid phosphatase (TRAP) are up‐regulated, giving rise to osteoclast differentiation and maturation.^12^, ^13^ The role of monocyte/macrophage lineage cell‐cell fusion for the process of osteoclastogenesis should not be neglected, because it lays a foundation for the eventual osteoclast maturation. This process relies on the dendrocyte‐expressed seven transmembrane protein (DC‐STAMP), osteoclast stimulatory transmembrane protein (OC‐STAMP) and CD9 to regulate the membrane fusion and multinucleation of osteoclasts. Moreover, in patients with Paget's disease, a variant of the DC‐STAMP gene‐promoting osteoclastogenesis has been identified. OC‐STAMP is another vital factor that positively modulates cell‐cell fusion independently of DC‐STMAP.^14^, ^15^, ^16^, ^17^

Inflammatory osteolysis is the common problem in clinic, driving bone degradation which affects the appendicular skeleton early in life and increases the fracture risk. Among the pathogenesis of inflammatory osteolytic diseases, lipopolysaccharide (LPS), an outer membrane component of Gram‐negative bacteria, was closely related to bone destruction and resorption. LPS stimulates the synthesis of several proinflammatory cytokines, such as IL‐1β, TNF‐α and IL‐6, which promote osteoclastogenesis, and ultimately, overactive osteoclasts lead to osteoclast‐related bone loss diseases.^18^, ^19^, ^20^ Therefore, regulating the differentiation of osteoclasts in inflammatory environment is also the key to alleviate LPS‐related inflammatory osteolysis. Moreover, several signalling pathways have been implicated in LPS‐induced inflammation, including NF‐ĸB, activator of transcription (STAT) and Janus Kinase（JAK). The signal transducer and STAT family member, STAT3, is particularly important as a part of a classical fundamental pathway involved in inflammation, which also participates in osteoclastogenesis; STAT3 activation results in a vital effect in various LPS‐induced models that leads to the binding of extracellularly released IL‐6 to its receptor, gp130. Overall, the decrease of LPS‐induced osteoclast maturation may significantly aid in slowing down the progression of inflammatory bone loss diseases. Furthermore, the IL‐6 receptor, gp130, can be stabilized by CD9 to activate STAT3.^21^, ^22^, ^23^, ^24^ These data imply that the inhibition of CD9 expression can indirectly impact IL‐6/gp130/STAT3 activation, which can serve as a new strategy for altering osteoclast behaviour under inflammatory conditions.

Formyl peptide receptors (FPRs) belong to the G protein‐coupled receptor family and consist of three isoforms, namely FPR1, FPR2/ALX and FPR3.^25^ FPRs have been identified in several cell types, such as neutrophils, monocytes and macrophages, and have multiple functions in vivo. Among these isoforms, several FPR2 agonists can suppress osteoclastogenesis. WKYMVm (Trp‐Lys‐Tyr‐Met‐Val‐D‐Met‐NH2), a synthetic peptide selected from peptide libraries, has been identified as a selective FPR2 agonist.^26^, ^27^ Interestingly, WKYMVm has demonstrated immunoregulatory ability under different physiological and pathological conditions through binding to the FPR2 with high affinity. Moreover, the anti‐inflammatory properties of this peptide have recently been recognized and its inhibitory activity on the production of inflammatory cytokines, such as interleukin (IL)‐1β and TNF‐α, has been demonstrated.^28^ This peptide has better affinity and low immunogenicity compared with the other FPR2 agonists. Therefore, it may have better efficiency to regulate osteoclastogenesis in inflammatory environment and better foreground to research the application of FPR2 in orthopaedics.

Based on the above rationale, we have been suggested that WKYMVm may inhibit osteoclast maturation under both normal and inflammatory conditions. Moreover, FPR2 may become the new target of regulating osteoclastogenesis and treating inflammatory osteolysis. To address this question, the effects of WKYMVm on osteoclastogenesis were investigated in RAW264.7 cells and BMMs. In addition, we aimed to expound the mechanism of WKYMVm‐mediated osteoclast differentiation and maturation. Our data showed that WKYMVm has inhibitory effects on RANKL and LPS‐induced osteoclast differentiation and maturation in vitro and LPS‐induced inflammatory bone loss in vivo. However, the inhibitory effect of WKYMVm could be hindered by WRWWWW (WRW4), an inhibitor of FPR2. Additionally, the molecular mechanism underlying these phenomena was based on WKYMVm‐mediated direct inhibition of NF‐ĸB and STAT3 signalling pathways and indirect inhibition of the CD9/gp130/STAT3 pathway. These findings imply that WKYMVm may have a negative regulatory effect on osteoclastogenesis and that FPR2 may serve as novel target in the future to regulate bone homeostasis.

## 2 MATERIALS AND METHODS

### 2.1 Materials and reagents

RAW264.7 cells were obtained from the American Type Culture Collection (Rockville, MD, USA). WKYMVm and WRWWWW (WRW4) with purity > 95% was synthesized by GL Biochem and was dissolved in acetonitrile to prepare stock concentrations of 0.1, 1, 2, 5 and 10 μmol/L. Dulbecco's Modified Eagle's Medium (DMEM), alpha minimum essential medium (α‐MEM) and foetal bovine serum (FBS) were purchased from Invitrogen‐Gibco. RANKL and soluble mouse recombinant M‐CSF were obtained from R&D Systems. The Acid Phosphatase, Leukocyte (TRAP) assay kit and Actin Cytoskeleton and Focal Adhesion (FAK) Staining Kit were purchased from Sigma (Sigma‐Aldrich) and Merck, respectively. The Cell Counting Kit‐8 (CCK‐8) was purchased from Beyotime Biotechnology. Anti‐phospho‐NF‐kBp65, anti‐NF‐kBp65, anti‐STAT3, anti‐phospho‐STAT3 (Ser727), anti‐CTSK, anti‐c‐Fos, anti‐NFATc1, anti‐CD9, anti‐gp130, anti‐IL6 and anti‐ß‐actin antibodies were purchased from Bioworld Technology. Mouse IL‐1β, IL‐6 and TNF‐α ELISA kits were procured from Novus.

### 2.2 Cell culture

RAW264.7 cells were cultured in a T25 culture flask containing complete culture medium composed of 90% DMEM and 10% FBS in an incubator set at a constant temperature of 37.8°C with 5% CO_2_. Bone marrow–derived macrophages (BMMs) were isolated mainly from the femurs and tibias of 4 to 6‐week‐old C57BL/6 mice. The complete procedure was performed according to the guidelines proposed by the Animal Care and Use Committee of the Third Military Medical University. Cells were incubated for 24 hours in α‐MEM supplemented with 30 ng/mL M‐CSF, 10% FBS, penicillin (100 units/mL) and streptomycin (100 mg/mL). Subsequently, non‐adherent cells were removed. After reaching confluence, adherent BMMs were cultured in a T25 culture flask with complete culture medium composed of 90% α‐MEM, 10% FBS, penicillin and streptomycin in an incubator at a constant temperature of 37°C with 5% CO_2_.

### 2.3 Cytotoxicity assay

WKYMVm cytotoxicity in RAW264.7 cells and BMMs was examined using a CCK‐8 kit. Both cell types were seeded in 96‐well plates at a density of 5 × 10^3^ per well and were treated with a range of WKYMVm concentrations (0.01, 0.1, 1 and 10 µmol/L) for 24 hours or 48 hours. Next, a CCK‐8 kit solution (10 µL/well) was added to the 96‐well plates, which were then incubated in the dark for 1 hour. Absorbance was detected at 450 nm and recorded by a microplate reader.

### 2.4 Osteoclastogenesis assay

BMMs and RAW264.7 cells were seeded in 96‐well plates overnight and then cultured in α‐MEM (BMMs) or DMEM (RAW264.7 cells) containing RANKL and M‐CSF with a range of WKYMVm doses or vehicle for 3 (RAW264.7 cells) or 5 days (BMMs). After the observation of multinucleated osteoclasts in the control group, the cells were fixed with 4% paraformaldehyde for 30 minutes. TRAP‐staining was performed in accordance with the manufacturer's instructions, and the cells were counted using an optical microscope (DMI 6000B; Leica Microsystems). TRAP‐positive multinucleated cells with 3 or more nuclei were identified as mature osteoclasts. For LPS‐induced osteoclastogenesis, RAW264.7 cells were cultured with RANKL (50 ng/mL) and M‐CSF (50 ng/mL) for 24 hours. Subsequently, RANKL and M‐CSF were replaced by LPS (100 ng/mL) and a range of WKYMVm concentrations or vehicle for 48 hours. After the cells were fixed and subjected to TRAP staining, TRAP‐positive multinucleated cells including 3 or more nuclei were counted under a light microscope. The quantitation of TRAP‐positive multinucleated cells (≥3 nuclei) was performed with ImageJ.

### 2.5 Actin Cytoskeleton and Focal Adhesion staining

RAW264.7 cells and BMMs were seeded in 96‐well plates at a density of 1 × 10^5^ cells per well. On the following day, complete medium containing RANKL (50 ng/mL), M‐CSF (50 ng/mL), and different WKYMVm concentrations and W4WR were added to the plates and incubated for 72 hours, until mature OCs were observed in the positive group. Subsequently, the cells were fixed with 4% paraformaldehyde at 37°C for 30 minutes and blocked with 3% BSA. Next, vinculin and F‐actin expression patterns were determined by incubating the cells first with anti‐vinculin or anti‐F‐actin primary antibodies for 1 hour and then with fluorescently conjugated secondary antibodies for 1 hour. Finally, the nuclei were stained with DAPI for 5 minutes. The data were recorded by fluorescent microscopy and quantitation of multinucleated cells was performed using ImageJ software.

### 2.6 RNA isolation and real‐time qPCR

To investigate marker gene expression in mature osteoclasts, quantitative real‐time polymerase chain reaction (qRT‐PCR) was used. BMMs and RAW264.7 cells were seeded in 6‐well plates at a density of 1 × 10^5^ cells per well and cultured in complete culture medium containing RANKL and M‐CSF, for 5 days (BMMs) and 3 days (RAW264.7) with or without a range of WKYMVm concentrations. After mature osteoclasts (OCs) were observed in the positive group, cells were lysed using Trizol buffer. Complementary DNA was synthesized from 1 μg total RNA from each sample using a reverse transcriptase kit. For real‐time PCR, 1 µg RNA was mixed with a PCR primer pair and SYBR green super mix. Glyceraldehyde 3‐phosphate dehydrogenase (GAPDH) was used as a control gene in this experiment.

### 2.7 Western blotting

BMMs and RAW264.7 cells were seeded in 6‐well plates at a density of 5 × 10^5^ per well and cultured with or without WKYMVm for 6 days (BMMs) and 3 days (RAW264.7 cells), until the formation of mature OCs in the positive group. Subsequently, cells were lysed in cell lysis buffer containing PMSF. Proteins were subjected to SDS‐PAGE and then electro‐transferred to PVDF membranes. Next, the membranes were blocked with 5% BSA for 2 hours and washed with TBS‐T buffer for 30 minutes. Specific primary antibodies against the proteins were incubated with the membranes in the specified dilutions for 12 hours at 4°C. Then, the membranes were incubated with horseradish peroxidase (HRP)‐conjugated secondary antibodies for 2 hours at room temperature and washed three times with TBS‐T buffer. Last, the Chemi Doc XRS+Imaging System (Bio‐Rad) was used to capture images of the protein bands, which were analysed with the ImageJ software. β‐Actin served as an internal control.

### 2.8 LPS‐induced calvarial osteolysis mouse model

A total of 20 male C57/BL6 mice, eight‐week‐old, were split randomly into four equal groups: sham, LPS, low‐dose WKYMVm (4 mg/kg bodyweight) and high‐dose WKYMVm (8 mg/kg bodyweight). The sham and LPS group were injected with PBS and LPS (5 mg/kg bodyweight), respectively. Predetermined WKYMVm doses were mixed with LPS and injected into the mice in combination. Injections were performed subcutaneously against the skull altogether 7 times with 48 hours intervals between applications. All mice were killed, and the calvariae were examined with micro–CT scan. After the micro–CT scans, all calvariae were fixed with 4% paraformaldehyde for 1 day and then decalcified in EDTA for 7 days. H&E staining and TRAP staining were performed, and the sections were observed under a microscope with 40‐100× magnification.

### 2.9 Intracellular reactive oxygen species detection

The ROS assay kit was used to detect the reactive oxygen species (ROS) levels in vitro. RAW264.7 cells were seeded in 96‐well plates at a density of 5 × 10^3^ cell/well incubated with RANKL (50 ng/mL) and M‐CSF (50 ng/mL) and treated with various concentrations of maleic acid for 3 days. Cells incubated with 2, 7‐dichlorodihydrofluorescein diacetate (DCFH‐DA) in 37°C without light for 30 minutes and then the images were observed under fluorescence microscopy.

### 2.10 Micro–CT scanning

High‐resolution micro–CT scan was performed on all mice (Quantum FX CT; Perkin Elmer). The CTAN program was used to estimate the calvaria bone volume per total volume (BV/TV).

### 2.11 Statistical analysis

All reported data are from at least three independent experiments. Data are presented as mean ± standard deviation (SD). Student's *t* test was used to analyse significant differences between two groups; the SPSS 22.0 software was utilized for all analyses. A *P* value lower than .05 was considered statistically significant.

## 3 RESULTS

### 3.1 WKYMVm‐mediated cytotoxicity in RAW264.7 cells and BMMs

**Figure 1:**
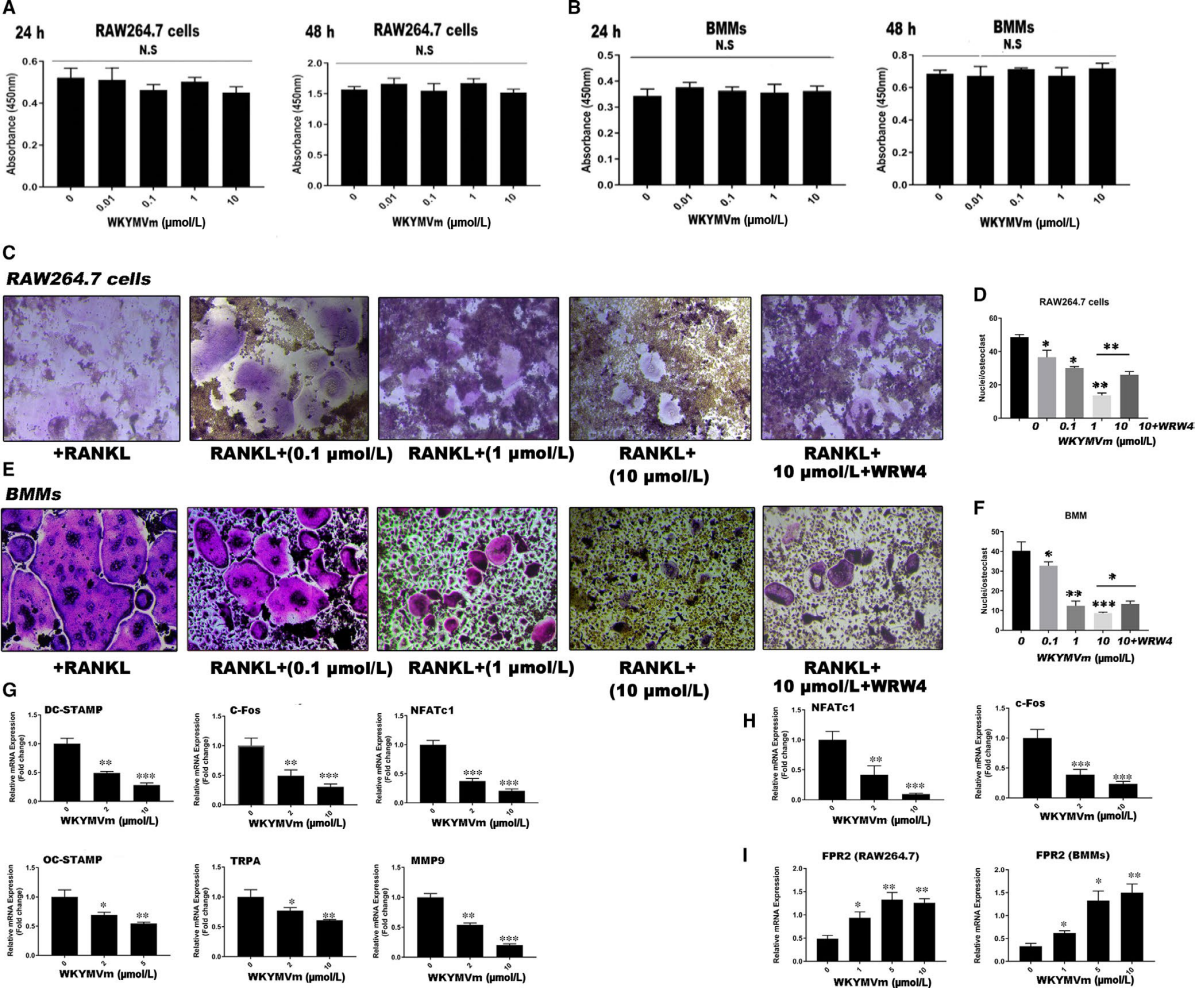
WKYMVm suppressed RANKL‐induced mature osteoclasts in vitro. A and B, CCK‐8 was used to assess RAW264.7 cell and BMM (C) RAW264.7 cells, treated with different WKYMVm concentrations (0.1, 1 and 10 µmol/L) or WRW4, were incubated with RANKL (50 ng/mL) and M‐CSF (50 ng/mL) for 72 h and then a TRAP‐staining was performed (scale bar, 200 µm). E, BMMs were incubated with RANKL (100 ng/mL) and M‐CSF (50 ng/mL) with or without WKYMVm and WRW4 until the appearance of mature osteoclasts in the control group. TRAP‐staining was performed and observed under a light microscope (scale bar, 200 µm). D and F, The area of TRAP‐positive multinucleated cells (>3 nuclei) was measured in each field using ImageJ software. Cells were cultured with RANKL (50 ng/mL), M‐CSF (50 ng/mL) and WKYMVm for 3 d (RAW264.7 cells) or 5 d (BMMs). The relative mRNA expression of (G) NFATc1, c‐Fos, DC‐STAMP, MMP9, OC‐STAMP and TRAP in RAW264.7 cells, and the relative mRNA expression of (H) NFATc1 and c‐Fos in BMMs were analysed using RT‐PCR. I, FPR2 in RAW264.7 and BMMs were analysed after treated with WKYMVm for 72 h by RT‐PCR. Gene expression was normalized to GAPDH. Data represent means ± SD. *P < .05, **P < .01 and ***P < .001 relative to RANKL‐induced controls

RAW264.7 cells and BMMs were incubated with a range of WKYMVm concentrations for 24 hours and 48 hours. To estimate the proliferation and cell viability of these cell types, a CCK‐8 kit was used. WKYMVm did not affect the proliferation of RAW264.7 cells (Figure 1A) and BMMs (Figure 1B) at concentrations <10 μmol/L. A high (10 μmol/L) and low (2 μmol/L) dose were selected to further investigate the effects of WKYMVm on mature OC formation.

### 3.2 WKYMVm suppressed RANKL‐induced mature osteoclast formation

To determine the effects of WKYMVm on osteoclast differentiation, BMMs and RAW264.7 cells were cultured in 96‐well plates with M‐CSF (50 ng/mL) and RANKL (50 ng/mL), and were treated with different WKYMVm concentrations for 120 hours (BMMs) and 72 hours (RAW264.7 cells) until multinuclear cells were observed in the control group. Among these, a group was treated by M‐CSF, RANKL, WKYMVm and WRW4. TRAP staining, examining TRAP activity, was used to estimate the effect of WKYMVm on OC differentiation and maturation. WKYMVm deceased the area occupied by and the number of mature OCs, as well as the nuclei per osteoclast number in RAW264.7 cells (Figure 1C,D) and BMMs (Figure 1E,F). However, slicing of FPR2 expression with WRW4 could attenuate the inhibitory effect of WKYMVm on osteoclastogenesis. Furthermore, osteoclast marker genes, including NFATc1, c‐Fos, DC‐STAMP, OC‐STAMP, MMP9 and TRAP, were measured in RAW264.7 cell‐derived (Figure 1G) and BMM‐derived osteoclasts (Figure 1H). BMMs and RAW264.7 cells were treated with RANKL (50 ng/mL), M‐CSF (50 ng/mL) and a range of WKYMVm concentrations; next, RT‐PCR was used to analyse osteoclast marker gene expression. WKYMVm apparently down‐regulated osteoclast marker gene expression relative to the control group, as demonstrated by TRAP‐staining. In addition, we detected the expression of FPR2 by RT‐PCR (Figure 1I). The results showed that WKYMVm could activate the expression of FPR2 whether in RAW264.7 cells or BMMs. Overall, the data indicate that WKYMVm inhibits RANKL‐induced differentiation of RAW264.7 cells and BMMs into mature OCs through FPR2.

### 3.3 WKYMVm repressed RANKL‐induced OC protein expression

**Figure 2:**
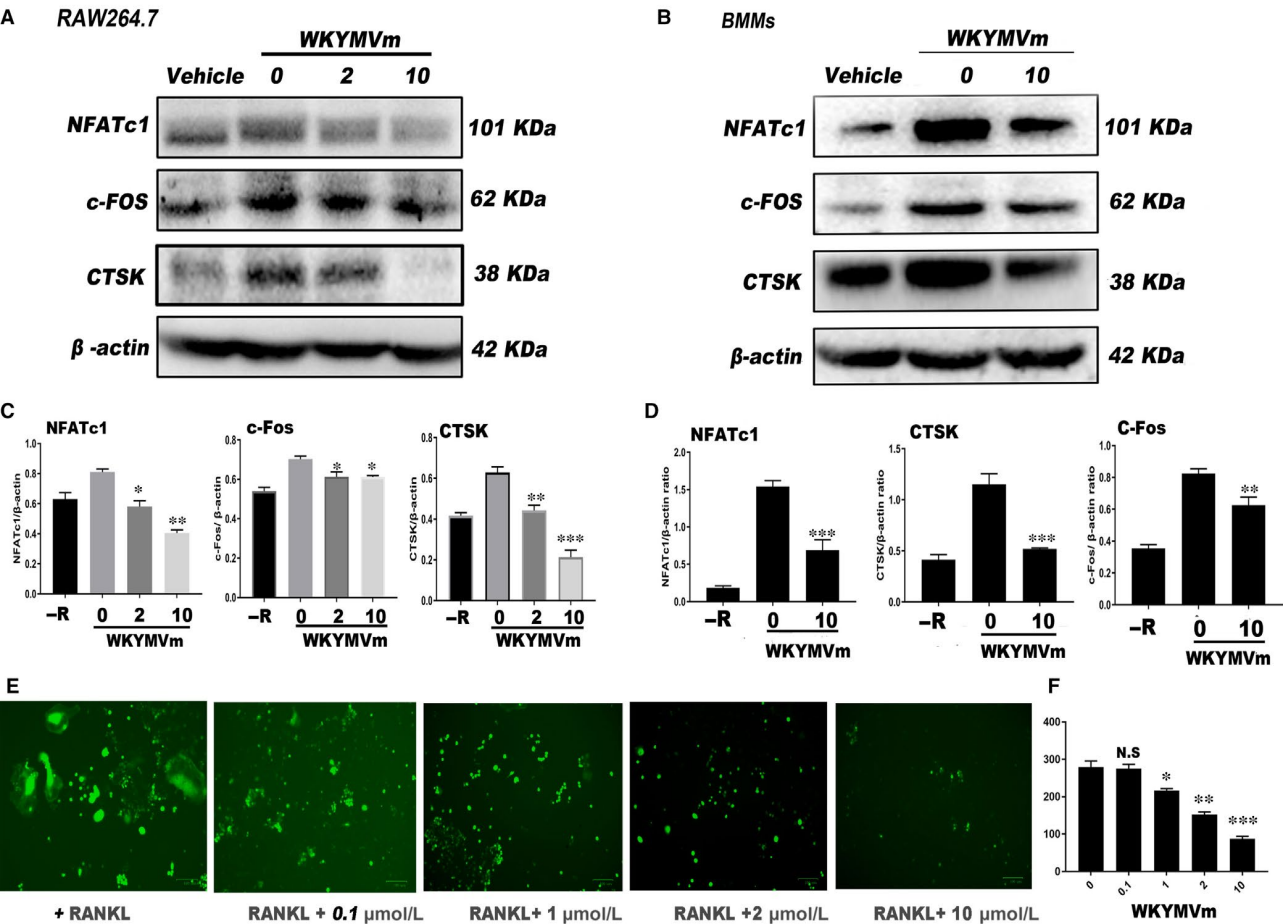
WKYMVm reduced the expression of OC maker proteins in vitro. Cells were seeded into 6‐well plates and cultured with predetermined WKYMVm concentrations for 3 d (RAW264.7 cells) or 5 d (BMMs). NFATc1, c‐Fos, CTSK and β‐actin protein levels were determined in (A) RAW264.7 cells and (B) BMMs by Western blot utilizing specific antibodies. Grey pixel value detection was used to analyse the relative expression of NFATc1, c‐Fos and CTSK, with β‐actin serving as a reference, in (C) RAW264.7 cells and (D) BMMs. E, The level of ROS was detected in RAW264.7 cells and (F) the quantification was measured. Data represent means ± SD. *P < .05, **P < .01 and ***P < .001 relative to RANKL‐induced controls

To investigate protein expression changes in osteoclasts treated with different WKYMVm concentrations, we performed Western blot after the formation of mature osteoclasts in the positive group. NFATc1 and c‐Fos, considered the most imperative transcription factors for the process of early osteoclast differentiation, and CTSK, a functional mature osteoclast gene, were detected by Western blot. Osteoclast protein expression was lower after WKYMVm treatment than in the control group (Figure 2A,B). WKYMVm down‐regulated the expression of NFATc1, c‐Fos and CTSK proteins in mature OCs (Figure 2C,D). In general, WKYMVm down‐regulated OC formation at the protein level.

### 3.4 WKYMVm decreased RANKL‐induced ROS level in RAW264.7 cells

As illustrated in Figure 2E, the level of ROS in RAW264.7 cells was determined after treating with WKYMVm. Apparently, WKYMVm could inhibit the intracellular ROS level and showed decreasing trend in dose‐dependent manner (Figure 2F).

### 3.5 WKYMVm alleviated OC resorption activity

**Figure 3:**
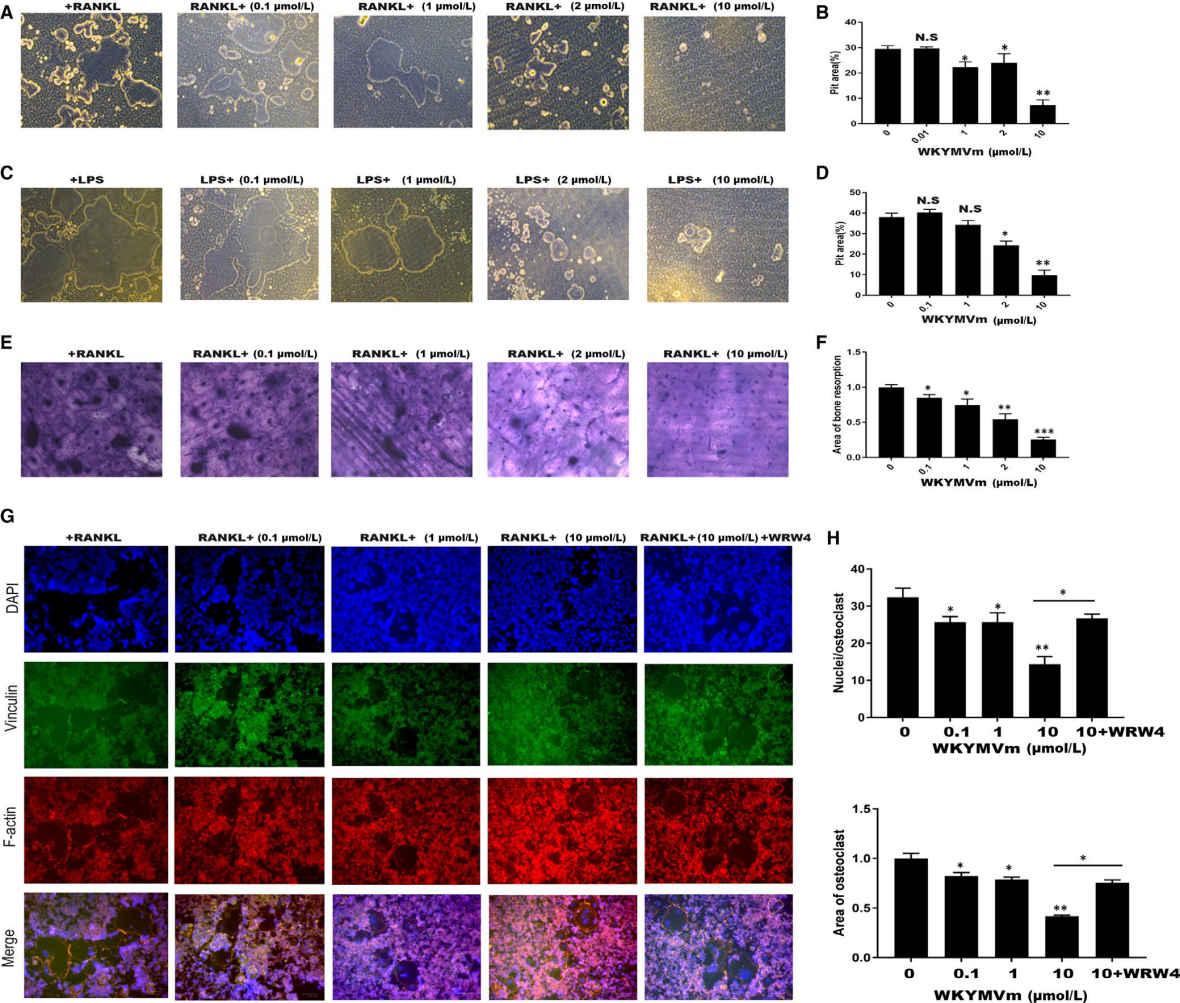
WKYMVm treatment decreased the resorption ability of mature osteoclasts in vitro*.* A, RAW264.7 cells, seeded into Osteo Assay surface plates, were incubated with RANKL (50 ng/mL) and M‐CSF (50 ng/mL) for 5 d with or without WKYMVm; next, osteoclasts were removed with sodium hypochlorite solution and captured using a light microscope (scale bar, 200 µm). B, The pit area percentage of Osteo Assay surface plates was measured with ImageJ software. C, Representative images of RAW264.7 cells in Osteo Assay surface plates cultured with LPS and different WKYMVm concentrations for 5 d (scale bar, 200 µm). D, The pit area percentage of Osteo Assay surface plates was measured using ImageJ software. E, RAW264.7 cells cultured in bovine bone slices and incubated with RANKL, CSF and predefined WKYMVm concentrations for 5 d (scale bar, 200 µm). F, The bone resorption area of bovine bone slices was calculated using ImageJ software. G, F‐actin, vinculin, and DAPI were observed by immunofluorescence microscopy (scale bar, 200 µm). H, Mature OC quantitation. Data represent means ± SD. *P < .05, **P < .01 and ***P < .001 relative to RANKL‐induced controls

Next, we used Osteo Assay surface plates and bovine bone slices, which were crucial for understanding the effect of WKYMVm on osteoclast resorption ability. RAW264.7 cells were seeded in Osteo Assay surface plates and on bovine bone slices, and were incubated with M‐CSF (50 ng/mL) and RANKL (50 ng/mL) containing different concentrations of WKYMVm for 120 hours (Figure 3A,E). Consistent with previous experiments, the erosion area was smaller in the WKYMVm‐treated groups than that in the group not treated with WKYMVm (Figure 3B,F). Next, we explored whether WKYMVm reduces the formation of the F‐actin ring. Actin Cytoskeleton and Focal Adhesion (FAK) staining was performed and observed under a fluorescence microscope (Figure 3G). WKYMVm inhibited F‐actin ring formation during RNAKL‐induced osteoclastogenesis through FPR2 (Figure 3H). In conclusion, osteoclast resorption activity was down‐regulated by WKYMVm.

### 3.6 WKYMVm inhibited LPS‐induced osteoclast differentiation in vitro

**Figure 4:**
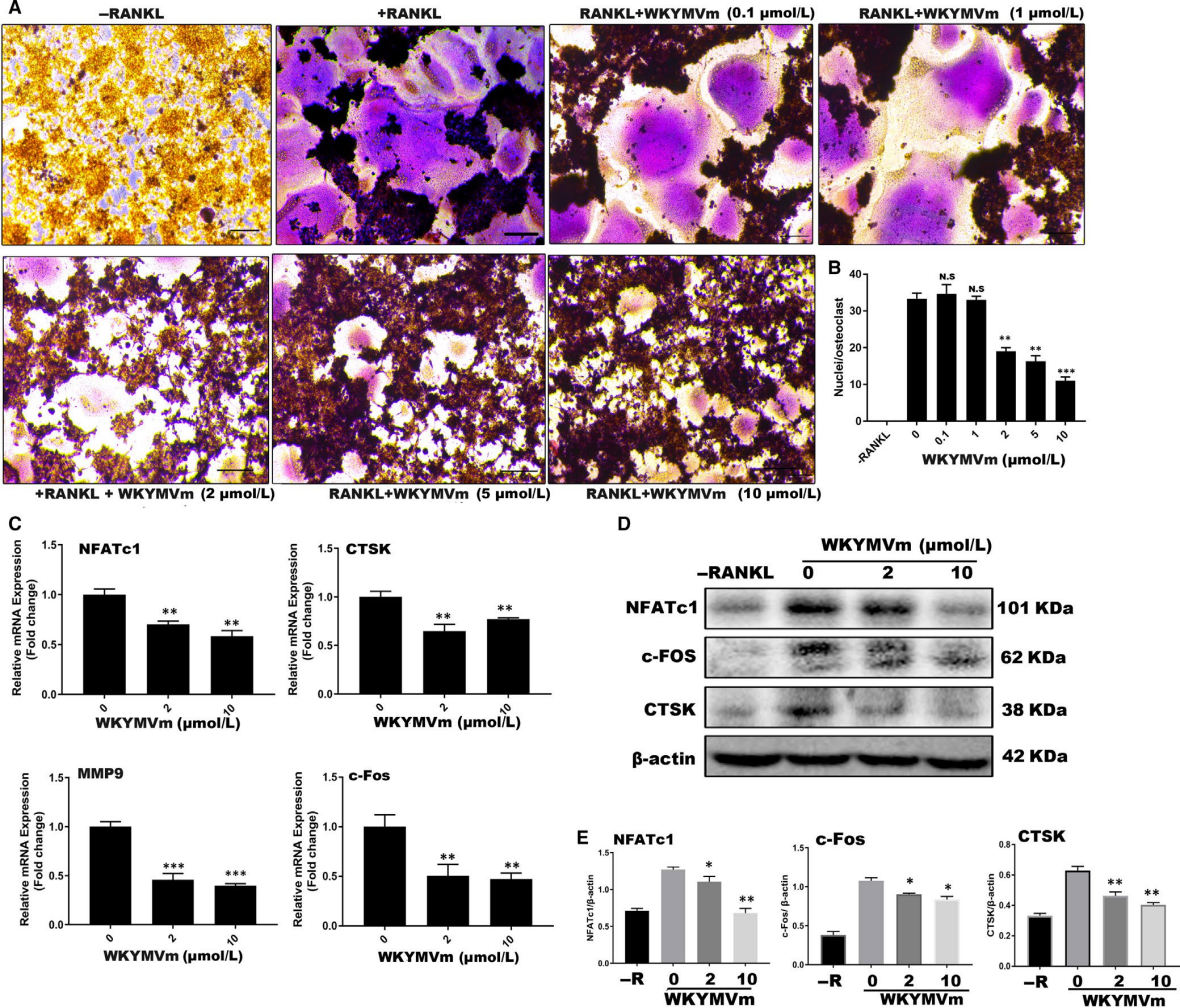
WKYMVm alleviated LPS‐induced osteoclastogenesis in vitro. A, RAW264.7 cells were pretreated with M‐CSF (50 ng/mL) and RANKL (50 ng/mL) for 24 h and subsequently cultured with LPS (100 ng/mL) alone or in combination with various WKYMVm doses for additional 72 h, until mature OCs were detected in the positive group; next, TRAP‐staining was performed and observed under a microscope (scale bar, 200 µm). B, The OC area (>3 nuclei) was estimated with ImageJ. C, RT‐PCR was used to analyse the mRNA expression of the markers NFATc1, c‐Fos, MMP9 and CTSK. Gene expression was normalized to GAPDH. D, RAW264.7 cells were treated with various WKYMVm concentrations in 6‐well plates for 3 d, and Western blot was carried out with specific antibodies against NFATc1, c‐Fos, CTSK and β‐actin. E, The intensity ratios of NFATc1, c‐Fos and CTSK relative to β‐actin were analysed by grey pixel value detection. Data represent means ± SD. *P < .05, **P < .01 and ***P < .001 relative to LPS‐induced controls

To test whether WKYMVm can suppress LPS‐induced differentiation of osteoclasts, we seeded RAW264.7 cells into 96‐well plates and cultured them with RANKL and M‐CSF for 24 hours. Subsequently, RANKL and M‐CSF were removed, and the cells were incubated with LPS and different WKYMVm concentrations for 48 hours. When mature OCs were formed in the positive group, TRAP‐staining was used to examine the TRAP activity (Figure 4A). The findings indicated that WKYMVm dose‐dependently prevents LPS‐induced osteoclastogenesis, and results were consistent with the non‐inflammatory environment (Figure 4B). Furthermore, we tested the expression of marker genes and proteins, demonstrating that WKYMVm indeed has the potential to inhibit osteoclastogenesis under inflammatory conditions (Figure 4C‐E). To further demonstrate that WKYMVm can prevent bone resorption in an inflammatory environment, an LPS‐induced osteoclastogenesis model was established using Osteo Assay surface plates (Figure 3C). WKYMVm inhibited osteoclast‐related resorption in an inflammatory environment (Figure 3D). Altogether, WKYVMm prevented osteoclastogenesis under inflammatory conditions.

### 3.7 WKYMVm suppressed osteoclastogenesis through the CD9/gp130/STAT3 and NF‐κB signalling pathways

**Figure 5:**
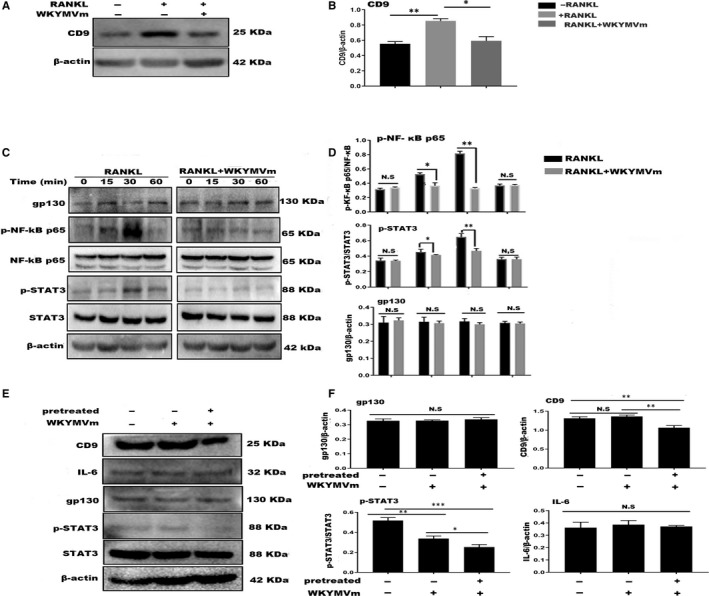
WKYMVm inhibited osteoclastogenesis directly via the NF‐κB and STAT3 signalling pathways and indirectly via CD9/gp130/STAT. A, RAW264.7 cells were cultured for 24 h with RANKL, M‐CSF and WKYMVm. Western blot was carried out with antibodies against CD9 and β‐actin. B, CD9 levels relative to β‐actin. C, RAW264.7 cells were treated with vehicle or WKYMVm for 2 h, followed by RANKL for 0, 15, 30 and 60 min. Western blot was carried out with antibodies against NF‐κBp65, p‐NF‐κBp65, STAT3, p‐STAT3, β‐actin and gp130. D, p‐NF‐κBp65 relative to NF‐κBp65 expression. STAT3 relative to p‐STAT3 levels. Gp130 relative to β‐actin expression. E, RAW264.7 cells were divided into 3 groups. One group was pretreated with RANKL, M‐CSF, LPS and WKYMVm for 24 h. The other two groups were pretreated with RANKL, M‐CSF and LPS, but not WKYMVm, for 24 h. After that, one group was treated with vehicle and the others—with WKYMVm for 2 h. Next, all groups were treated with RANKL for 30 min. The cell lysates were analysed using Western blotting for CD9, IL‐6, gp130, STAT3, p‐STAT3 and β‐actin. F, Quantification of IL‐6, gp130 and CD9 relative to β‐actin, and p‐STAT3 relative to STAT3. Data represent means ± SD. *P < .05, **P < .01 and ***P < .001 relative to LPS‐induced controls

CD9, an early fusion molecule, stabilizes gp130 by preventing its ubiquitin‐dependent lysosomal degradation to promote STAT3 activation in glioma stem cells. Therefore, we investigated CD9 expression after treating RAW264.7 cells with M‐CSF, RANKL and predefined WKYMVm doses for 24 hours. WKYMVm repressed CD9 expression after 24 hours, inhibiting preosteoclast membrane fusion (Figure 5A,B). To explore WKYMVm effects on the STAT3 and NF‐ĸB signalling pathways, we seeded RAW264.7 cells in 6‐well plates overnight and then starved the cells with DMEM without FBS for 12 hours. Next, the cells were incubated with vehicle or WKYMVm (10 µmol/L) and then with RANKL and M‐CSF for 0, 15, 30 and 60 minutes. Western blot was carried out with NF‐kB, p‐NF‐kB, STAT3, p‐STAT3 and β‐actin specific antibodies. WKYMVm hindered STAT3 and NF‐ĸB phosphorylation, indicating that WKYMVm inhibits osteoclasts directly through these pathways (Figure 5C,D). Regarding the prevention of CD9 expression by WKYMVm, we have been suggested that WKYMVm can restrain STAT3 activity indirectly through the CD9/gp130/STAT3 pathway. We pretreated RAW264.7 cells with M‐CSF, RANKL and LPS with or without WKYMVm for 24 hours and determined CD9 expression differences. As WKYMVm inhibits CD9 expression rapidly, CD9 levels were lower in cells pretreated with WKYMVm than in the groups not treated with the peptide. Subsequently, the cells were cultured with DMEM without FBS for 12 hours to bring phospho‐STAT3 expression to an equivalent level in all cells. Next, the cells were incubated with a vehicle and WKYMVm (10 μmol/L) followed by RANKL and M‐CSF for 30 minutes. Although the gp130 and IL‐6 expression did not differ significantly among the groups, the STAT3 phosphorylation level was lower in the group pretreated with WKYMVm for 24 hours than in the other two groups (Figure 5E,F). Based on these results, we concluded that WKYMVm reduces phospho‐STAT3 levels directly and can rapidly suppress the level of CD9, but not gp130, to inhibit CD9 and gp130 combination, which can down‐regulate STAT3 phosphorylation.

### 3.8 WKYMVm protected against LPS‐induced bone loss in vivo

**Figure 6:**
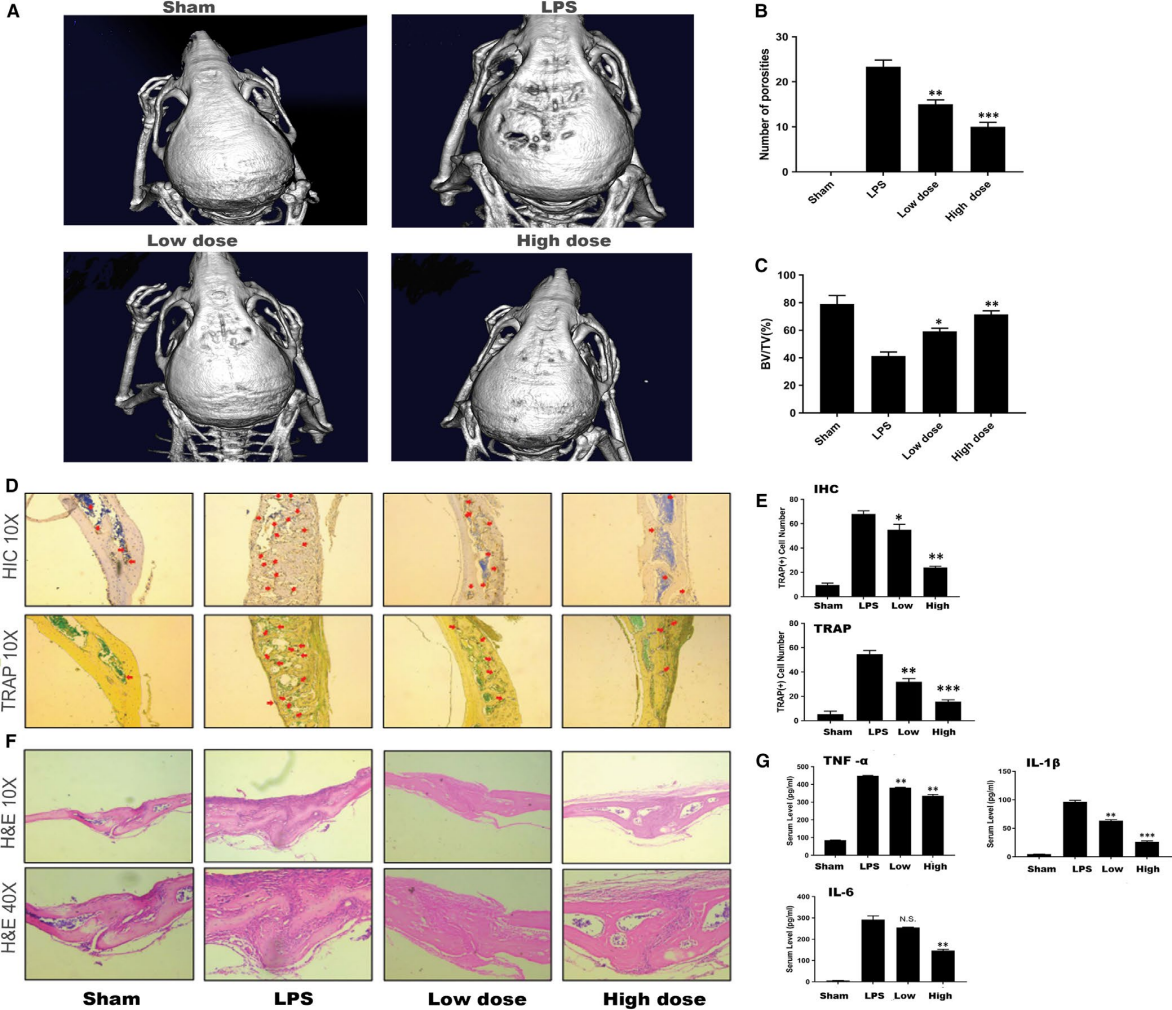
WKYMVm inhibited osteolysis in vivo. A, Micro–CT scanning and 3D reconstruction of calvaria outer surface in the sham, LPS, LPS with low‐dose WKYMVm and LPS with high‐dose WKYMVm groups. B and C, The BV/TV% and the porosity number of whole calvariae were measured by quantitative analysis. D and E, Representative images of cranial sections stained with TRAP and immunohistochemistry of TRAP. Red arrows indicate TRAP‐positive cells. The TRAP‐positive cells were measured by quantitative analysis. F, Mouse calvariae stained with H&E from the sham, LPS, LPS with low‐dose WKYMVm and LPS with high‐dose WKYMVm groups. G, In mouse serum, TNF‐α, IL‐1β and IL‐6 expression levels were detected by ELISA. Data represent means ± SD. *P < .05, **P < .01 and ***P < .001 relative to LPS‐induced controls

To investigate the practical applicability of this mechanism in vivo, an LPS‐induced osteolytic model was established in male C57/BL6J mice. We injected LPS with or without WKYMVm subcutaneously into the calvariae of the vehicle group and WKYMVm group to introduce these models, as well as PBS into the sham group to create a blank control for 14 days. The bone loss extent was estimated by micro–CT scanning and 3D image reconstruction. According to the results, the bone mass of the LPS group was significantly reduced compared with the ones of the sham and WKYMVm‐treated groups (Figure 6A). To further investigate the anti‐inflammatory effect of WKYMVm, we analysed the bone parameters of these groups, which demonstrated the protective capability of WKYMVm in the LPS‐induced osteolytic model. The data indicated an apparent decrease in the BV/TV of the LPS group with more porosity in comparison with the WKYMVm‐treated group (Figure 6B,C). Furthermore, the result of H&E histological staining was consistent with the results of the micro–CT scans (Figure 6F). The results of TRAP staining in histology and immumohistochemical staining of TRAP were in keeping with the effect in vitro(Figure 6D,E). ELISA was used to analyse the expression of IL‐6, TNF‐ α and IL‐1β (Figure 6G). WKYMVm inhibited proinflammatory cytokine expression. Therefore, we concluded that WKYMVm has a therapeutic potential to prevent LPS‐induced bone loss in vivo.

## 4 DISCUSSION

WKYMVm, a strong FPR2 agonist, was recently reported to have multiple effects on cell differentiation.^25^ As a major FPR2 agonist, WKYMVm exhibits anti‐inflammatory and anti‐tumour effects. However, its role in osteology has rarely been investigated.^31^, ^32^ Other FPR2 agonists have previously been reported to inhibit osteoclastogenesis.^33^, ^34^ Accordingly, we have been suggested that WKYMVm, another FPR2 agonist, also suppresses osteoclastogenesis under both normal and inflammatory condition. In this study, we demonstrated that WKYMVm negatively regulates osteoclast differentiation and maturation in RNAKL‐induced or LPS‐induced osteoclastogenesis through FPR2 in vitro. Furthermore, WKYMVm reduced the bone resorption activity of mature osteoclasts. Mechanistic research revealed that WKYMVm inhibits osteoclastogenesis directly through reducing the phosphorylation of the NF‐κBp65 and STAT3 signalling pathways. Furthermore, the CD9/gp130/STAT3 pathway was also indirectly negatively regulated by WKYMVm.

It is broadly accepted that osteoclast maturation is essential for bone resorption, which is the main pathogenetic mechanism for osteoclast‐related bone loss diseases.^29^, ^30^ Our findings indicated that the biological behaviour of osteoclasts is modulated by WKYMVm. First, WKYMVm significantly suppressed the expression of NFATc1 and c‐Fos during osteoclast maturation, leading to a reduction in osteoclast number, average nuclei number and osteoclastogenesis area in vitro. Activated NFATc1 and c‐Fos are also responsible for the expression of osteoclast specific genes, including TRAP, CTR, CTSK and MMP9, ultimately promoting osteoclast differentiation, proliferation and maturation. Second, WKYMVm reduced the mRNA expression of DC‐STAMP and OC‐STAMP to hinder membrane fusion and multinucleation. Additionally, WKYMVm lowered the protein expression of CD9 to further inhibit F‐actin ring formation. Under physiological conditions, the fusion between monocyte/macrophage lineage cells is also a vital step in the differentiation and maturation of osteoclast precursors into mature multinucleated osteoclasts. The cell‐cell fusion can be divided into four main cellular phases: (1) pre‐fusion programming of the cells, (2) chemotaxis, cellular adhesion and cytoskeletal rearrangements, (3) membrane fusion and multinucleation and (4) post‐fusion macrophage reprogramming. DC‐STAMP and OC‐STAMP, which are essential for cell‐cell fusion, participate in step 3.^39^ Another fusion‐related molecule in this step is CD9, a tetraspanin protein that controls cell‐cell fusion as an OC‐STAMP partner.^35^ Therefore, reduced gene expression demonstrated that osteoclast biological behaviour can be changed by WKYMVm to suppress osteoclastogenesis.

In addition, MMP9 and CTSK, two of the osteoclastogenic genes, have been previously critically implicated in the resorption activity of mature osteoclasts. Specifically, MMP9 mediates osteoclast adhesion and migration to resorption sites. Furthermore, MMP9, in combination with CTSK, plays an irreplaceable role in the degradation of ECM protein and type I collagen, which are imperative bone components.^39^, ^40^ Accordingly, osteoclast resorption activity can be reduced by inhibiting MMP9 and CTSK expression. The results from our study supported the notion that WKYMVm inhibits the expression of both MMP9 and CTSK to decrease the resorption capacity of osteoclasts.

Under inflammatory conditions, osteoclastogenesis can be promoted. For example, preosteoclasts treated with RANKL prior to LPS induction can stimulate osteoclastogenesis in inflammatory processes. We found that WKYMVm hinders osteoclastogenesis in LPS‐induced osteoclasts in vitro and LPS‐induced inflammatory bone loss model in vivo. In LPS‐simulated inflammatory processes, TNF‐α, IL‐6 and IL‐1β are classical inflammatory cytokines and are seen as the stimuli promoting osteoclastogenesis. These proinflammatory cytokines activate several inflammatory‐related pathways, including the NF‐κB, MAPK and STAT signalling pathways, resulting in the nuclear translocation of NFATc1 and c‐Fos to influence osteoclast differentiation.^18^, ^19^, ^20^, ^21^, ^22^, ^23^ We found that WKYMVm suppresses IL‐6, TNF‐β and IL‐1α synthesis in vivo; however, IL‐6 was not changed by WKYMVm in vitro. This inconsistency may result from the complicated in vivo environment. Moreover, LPS‐induced osteoclastogenesis was suppressed by WKYMVm, indicated by osteoclast number, average nuclei number or bone resorption ability, and consistent with the results of RANKL‐induced osteoclastogenesis. These results suggest that WKYMVm can restrain osteoclastogenesis under both normal and inflammatory conditions.

**Figure 7:**
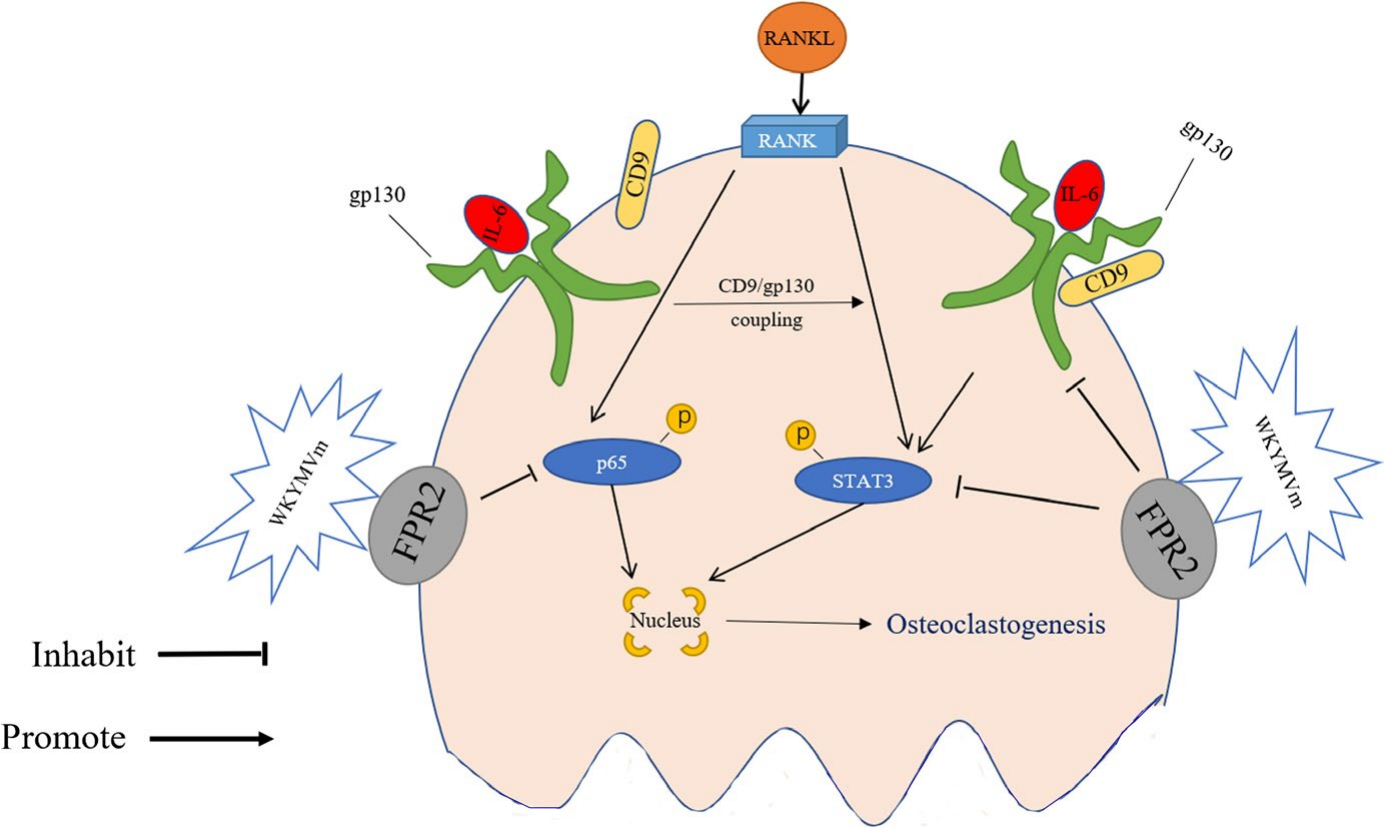
Schematic diagram of the inhibitory effect of WKYMVm on osteoclast formation and bone resorption activity

Additionally, during the exploration of the underlying mechanism, we found that WKYMVm suppresses osteoclastogenesis directly through NF‐κB and STAT3 and indirectly through CD9/gp130/STAT3. The NF‐κB signalling pathway is considered essential for osteoclast differentiation and maturation. NF‐κB promotes the nuclear translocation of NFATc1 and c‐Fos, leading ultimately to osteoclastogenesis.^6^, ^23^ In our study, NF‐κB phosphorylation was reduced by WKYMVm. Interestingly, STAT3, a STAT family member, is a part of a classic chronic pathway related to inflammation, which also participates in osteoclastogenesis. STAT3 can be activated by IL‐6 in cells expressing IL‐6R and gp130. Previous studies have demonstrated that IL‐6 recruits gp130 by binding to its receptor, the membrane‐bound IL‐6 receptor (mIL‐6R), to activate STAT3.^36^, ^37^, ^38^ Moreover, CD9 stabilizes gp130 to activate STAT3 in glioma stem cells by preventing its ubiquitin‐dependent lysosomal degradation. ^24^ Based on these findings, we have been suggested that inhibition of CD9 expression may also interfere with the IL6/gp130/STAT3 pathway under inflammatory conditions to alter osteoclastogenesis behaviour. Interestingly, in our study, WKYMVm could repress CD9 to inhibit cell‐cell osteoclast fusion. Therefore, we have been suggested that WKYMV reduces STAT3 phosphorylation through hindering the combination of CD9 and gp130. Previously, several other FPR2 agonists have been analysed and have shown different relationship to that of STAT3. Thus, LX4 was a positive regulator of STAT3 activation, whereas SAA had no effect on the phosphorylation of STAT3. In our mechanistic study, we showed that WKYMVm represses STAT3 phosphorylation to directly inhibit osteoclastogenesis. This paradox may result from the different cell types utilized in the experiments or from differences in FPR2 activity; this suggests that FPR2 has pleiotropic regulatory potential under different physiological and pathological conditions, and under the influence of different FPR2 agonists on IL‐6, gp130 and CD9. Moreover, WKYMVm did not reduce IL‐6 expression in line with previous studies, and gp130 was also not influenced by WKYMVm. Although WKYMVm did not affect IL‐6 and gp130 expression in LPS‐induced osteoclastogenesis, our results demonstrated that WKYMVm can still affect the STAT3 signalling pathway through repressing CD9, but not gp130, expression to reduce CD9 and gp130 binding. Altogether, our data indicate that WKYMVm has the ability to negatively modulate osteoclastogenesis directly through NF‐κB and STAT3 and indirectly through CD9/gp130/STAT3. The schematic diagram of inhibition of osteoclast differentiation and formation by WKYMVm was shown in Figure 7.

In conclusion, our study proved that WKYMVm suppresses RANKL and LPS‐induced osteoclastogenesis through direct and indirect inhibition of STAT3 and CD9/gp130/STAT3 activity, respectively. And the down‐regulatory effect of WKYMVm could be attenuated by slicing FPR2 expression. These data may outline a new mechanism for the regulation of osteoclast maturation and function, and FPR2 may represent a novel future target to moderate bone homeostasis.

## CONFLICT OF INTEREST

The authors have no conflicts of interest to declare.

## AUTHOR CONTRIBUTIONS

J. Hu, X. Han and L. Li completed in vitro studies; J. Hu and X. Li performed the in vivo studies; J. Hu, Z. Yang and Y. Chen analysed the data; J. Hu and Y. Chen wrote the paper; J. Hu and Q. He designed this study; and J. Hu and Q. He performed final manuscript.

## Data Availability

The data used to support the findings of this study are available from the corresponding author upon reasonable request.
