# Correction to ‘The Protective Effect of WKYMVm Peptide on Inflammatory Osteolysis Through Regulating NF‐κB and CD9/gp130/STAT3 Signalling Pathway’

**DOI:** 10.1111/jcmm.71282

**Published:** 2026-07-11

J. Hu , X. Li , Y. Chen , et al., “The Protective Effect of WKYMVm Peptide on Inflammatory Osteolysis Through Regulating NF‐κB and CD9/gp130/STAT3 Signalling Pathway,” Journal of Cellular and Molecular Medicine 24, no. 2 (2020): 1893–1905. 10.1111/jcmm.14885.31837208 PMC6991638

In the published article, an error was identified in Figure 3E, which may have been caused by the incorrect use of panels during figure compilation. The corrected Figure 3E is provided below. This correction does not affect the results or conclusions of the article.

Corrected Figure 3E:

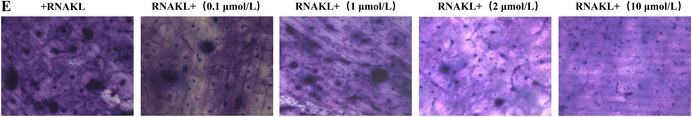

The authors apologise for these errors and for any inconvenience caused.

